# Timed exercise modulates inter-coupling strength between evening and morning oscillators in mice

**DOI:** 10.1038/s44323-026-00075-3

**Published:** 2026-03-27

**Authors:** Nagomi Miyagi, Noriko Matsuura, Yujiro Yamanaka

**Affiliations:** 1 Department of Education, Hokkaido University, Sapporo, Japan; 2 Laboratory of Life & Health Sciences, Faculty of Education and Graduate School of Education, Hokkaido University, Sapporo, Japan; 3 Research and Education Center for Brain Science, Hokkaido University, Sapporo, Japan

**Keywords:** Neuroscience, Physiology

## Abstract

The present study examined the effect of daily exposure to a novel cage equipped with a running wheel (NCRW) at two different times of day on the coupling strength between the evening (E) and morning (M) oscillators in nocturnal rodents. Adult male C57BL/6 mice (2–4 months old) were individually housed in cages without running wheels and maintained under a 12:12 h light-dark (LD) cycle with ad libitum access to food and water. Daily 3‑h exposure to the NCRW was conducted on five consecutive days per week for three weeks (19 days total) at either ZT12 (zeitgeber time: ZT, ZT12 = dark onset of the LD cycle) or ZT21. To assess effects on circadian behavioral rhythms, we performed three experiments: (1) measurement of the free-running period under constant darkness (DD), (2) assessment of the reentrainment rate to an 8-h phase advance of the LD cycle, and (3) analysis of phase shifts induced by a single 8-h advanced LD cycle. Exposure to NCRW at ZT12 significantly shortened the free-running period and facilitated reentrainment of activity onset to the advance of the LD cycle. In contrast, exposure to the NCRW at ZT21 lengthened the free-running period and significantly decelerated reentrainment of activity onset compared with the control condition. These results suggest that exposure to NCRW at ZT12 enhances the coupling strength of the E oscillator to the M oscillator, whereas exposure to NCRW at ZT21 enhances the coupling strength of the M oscillator to the E oscillator. These findings indicate that timed exercise under the LD cycle phase dependently modulates inter-oscillator coupling strengths and light-induced phase resetting, thereby influencing the regulation of activity onset and offset in nocturnal rodents.

## Introduction

Circadian rhythms in mammals are orchestrated by the central pacemaker located in the suprachiasmatic nucleus (SCN) of the hypothalamus^1^. The SCN entrains to environmental light-dark (LD) cycles via photic input from retinal ganglion cells^2,3^. Although the LD cycle is the primary zeitgeber for the central pacemaker in the SCN, timed physical activity, such as timed wheel running activity^4–6^ and forced treadmill running^7,8^, can act as a potent nonphotic zeitgeber under constant darkness (DD). Previous studies have demonstrated that free access to a running wheel shortens the free-running behavioral period in both rats^9^ and mice^10^. Moreover, timed exercise induced by exposure to a new cage with a running wheel (NCRW) can entrain both behavioral rhythms and clock gene expression in the SCN and some peripheral tissues under DD in mice^6,11^.

Regarding the effects of timed exercise on circadian behavioral rhythms, Mistlberger and Holmes showed that restricted wheel access, achieved by locking or unlocking an RW at specific times of day under the LD cycle, altered the free-running rhythm of wheel-running activity under DD. These findings suggested that timed exercise during the early or late subjective night shortened or lengthened the free-running period under DD^12^. However, that study focused exclusively on activity onset as the circadian behavioral marker.

In the present study, to distinguish the effects of timed exercise from the influence of spontaneous wheel-running activity, mice were housed in a cage without an RW except during scheduled exercise. Furthermore, to investigate the coupling dynamics between the evening (E) and morning (M) oscillators in the SCN, we analyzed both the activity onset and offset.

In nocturnal rodents, activity duration is thought to be regulated by two coupled oscillators, referred to as the E and M oscillators, which separately regulate activity onset and offset^13–15^. Previous studies have suggested that the magnitude of light-induced phase shifts, evaluated on the next cycle after a light pulse, differs between activity onset and offset, indicating that the E and M oscillators have different light sensitivities in terms of the immediate phase‑response curve (PRC)^16–18^. In addition, it has been demonstrated that the E oscillator has an endogenous period shorter than 24 h, whereas the M oscillator has a period longer than 24 h. Based on the E and M oscillator model, activity onset and offset reach and maintain a steady‑state phase relationship under constant conditions, during which the free‑running period is determined by bidirectional coupling between the E and M oscillators. For example, strengthening one oscillator can influence the phase and period of the other.

To elucidate the effects of timed exercise on the coupling force between these two oscillators, it is necessary to assess both the period and phase of activity onset and offset when exercise is administered at specific times of day.

Regarding non-photic phase shifting and entrainment by timed exercise, when steady-state entrainment is established through daily exposure to NCRW under DD conditions, activity duration—defined as the interval between onset and offset—is shortened in mice entrained to the scheduled exposure to NCRW^6,11^. After an abrupt shift of the LD cycle, the activity onset and offset of locomotor activity show asymmetric reentrainment, in which activity offset is largely advanced by the shifted light period, whereas the activity onset gradually shifts^19^. Exposure to the NCRW in the middle of the subjective day accelerates the reentrainment of the circadian rhythm of activity onset in mice^19,20^. These findings suggest that daily exposure to the NCRW can modulate the coupling force and light-induced phase shifts of the E and M oscillators. Although many previous studies have examined the phase‑shifting effects of exercise under DD^4,6,7^ or after an abrupt shift of the LD cycle^19–22^, much less is known about how timed exercise interacts with the E and M oscillators entrained to the LD cycle. By evaluating both activity onset and offset, the present study addresses this gap and provides a framework for assessing how timed exercise modulates coupling strengths between the E and M oscillators.

To study this hypothesis, we examined whether timed exercise by exposure to NCRW at two different times of day under the LD cycle alters the subsequent free-running period under DD and light-induced phase shifts of activity onset and offset in mice.

## Results

### Experiment 1: circadian period and phase angle difference of entrainment

**Fig. 1:**
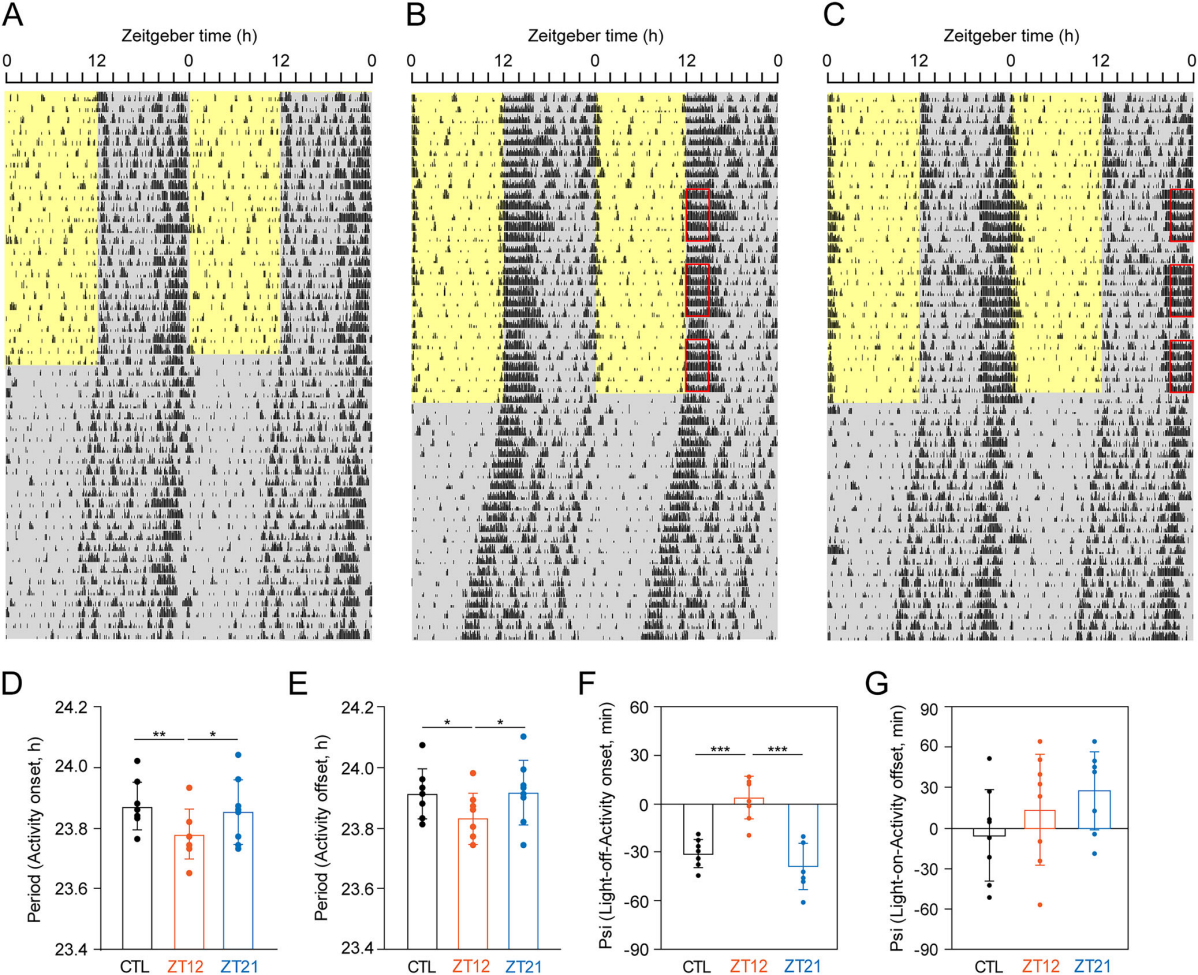
Representative actograms of locomotor activity from mice subjected to exposure to a new cage with a running wheel (NCRW) under a light-dark (LD) cycle, followed by release into constant darkness (DD). Representative double-plot actograms of locomotor activity from the same mouse under three different conditions during an LD cycle: control (**A**), exposure to NCRW at ZT12 (**B**), and exposure to NCRW at ZT21 (**C**). Red squares indicate the 3-h NCRW exposure at ZT12-ZT15 (**B**) or ZT21-ZT0 (**C**). Yellow and gray areas indicate light and dark periods, respectively. Square bars indicate the mean ± SD of the free-running period of activity onset (**D**) and offset (**E**) under DD; small circles represent individual values. Phase angle differences (Psi) between dark onset and activity onset (**F**) and between light onset and activity offset (**G**). Square bars and small circles indicate mean and individual values, respectively. In (**D**–**G**), black, orange, and blue bars and circles indicate the data from the control (CTL), NCRW exposure at ZT12, and NCRW exposure at ZT21, respectively. Asterisks indicate significant differences determined by Tukey’s post hoc test following one-way repeated-measures ANOVA. * *P* < 0.05, ** *P* < 0.01, *** *P* < 0.001.

Figure 1 shows representative double-plotted actograms of locomotor activity from the same mouse under three different conditions: control (Fig. 1A), and exposure to the NCRW at ZT12 (Fig. 1B), and ZT21 (Fig. 1C).

Repeated measures ANOVA revealed significant differences in the free-running period among the three conditions (activity onset: *F* = 6.78, *P* = 0.01; activity offset: *F* = 5.03, *P* = 0.02). Under DD, the free-running periods of activity onset and offset were significantly shortened in mice exposed to the NCRW at ZT12 (activity onset: 23.78 ± 0.08 h, Fig. 1D; activity offset: 23.83 ± 0.08 h, Fig. 1E) compared to those in the control condition (activity onset: 23.87 ± 0.08 h, *P* = 0.0001, Fig. 1D; activity offset: 23.91 ± 0.08 h, *P* = 0.044; Fig. 1E) and the exposure at ZT21 (activity onset: 23.85 ± 0.11 h, *P* = 0.039, Fig. 1D; activity offset: 23.92 ± 0.11 h, *P* = 0.035, Fig. 1E). No significant difference in free-running period was observed between the control and exposure at ZT21 (Fig. 1D, E).

Repeated measures ANOVA revealed a significant difference in the phase angle difference (ψ) between the activity onset and dark onset in the LD cycle among the three conditions (*F* = 24.47, *P* < 0.0001), but not in the ψ between activity offset and light onset in the LD cycle (*F* = 1.516, *P* = 0.253). The ψ between activity onset and dark onset was significantly more positive in mice exposed to the NCRW at ZT12 (3.7 ± 13.4 min) than in those in the control (−31.1 ± 8.6 min, *P* = 0.0003) and the exposure at ZT21 (−38.8 ± 14.3 min, *P* < 0.0001, Fig. 1F) conditions. The ψ between activity offset and light onset was −5.6 ± 34.2 min in the control, 13.5 ± 41.2 min in the exposure to NCRW at ZT12, and 27.3 ± 29.0 min in the exposure at ZT21, indicating greater interindividual variability (standard deviation) compared with the ψ between activity onset and dark onset (Fig. 1G).

### Experiment 2. Reentrainment of circadian behavioral rhythms to an 8-h advanced LD cycle

**Fig. 2:**
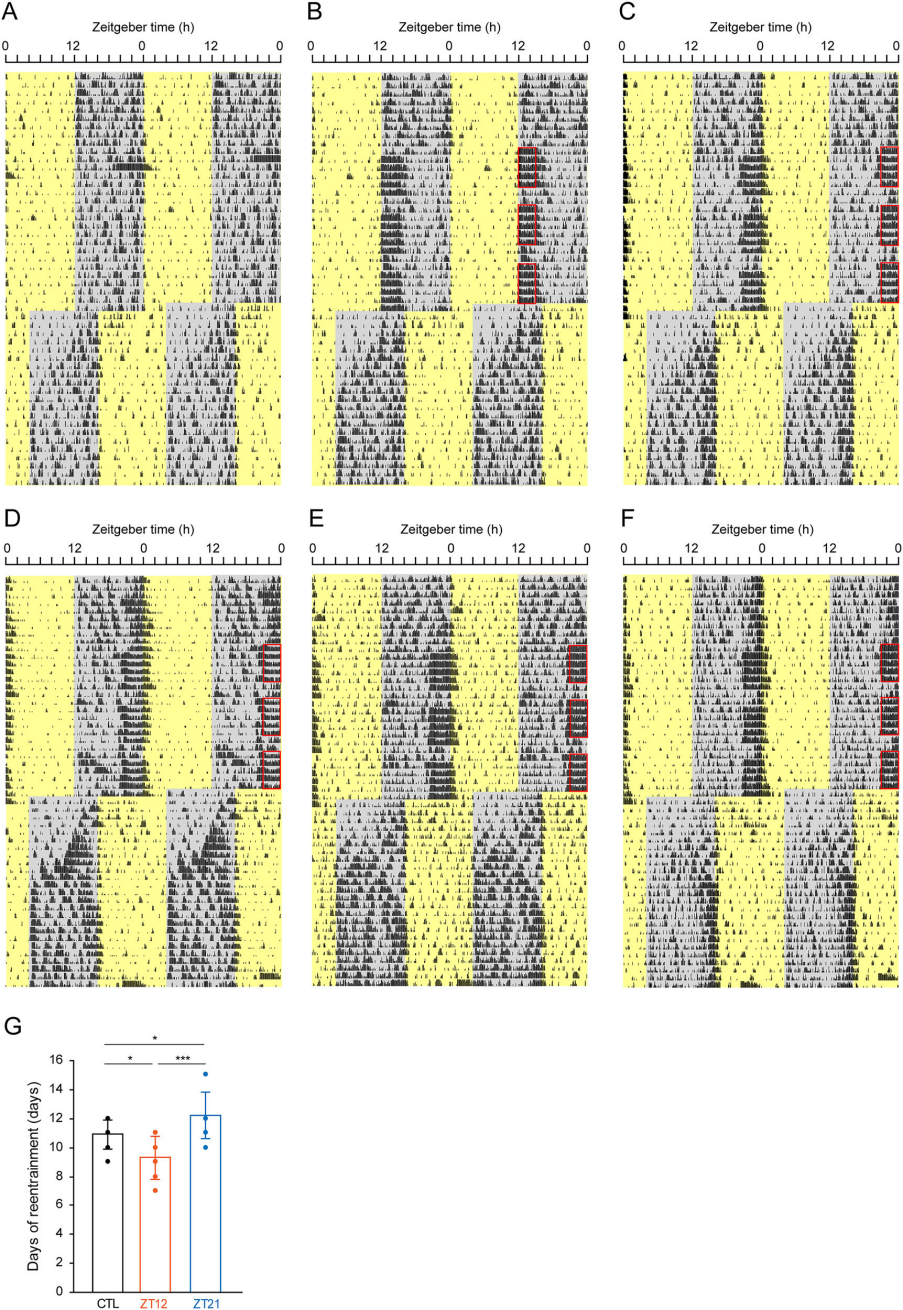
Representative actograms of locomotor activity from mice exposed to a new cage with a running wheel (NCRW) under a light-dark (LD) cycle followed by an 8-h advance of the LD cycles. Representative double-plotted actograms of locomotor activity from the same mouse under three different conditions during an LD cycle: control (**A**), exposure to NCRW at ZT12 (**B**), and exposure to NCRW at ZT21 (**C**). Red squares indicate the 3-h NCRW exposure periods at ZT12-ZT15 (**B**) or ZT21-ZT0 (**C**). Yellow and gray areas indicate light and dark periods, respectively. **D**–**F** Representative double-plotted actograms in mice exposed to NCRW at ZT21 showing slow phase advances of activity onset or delay shift of some activity bouts. **G** indicate the mean and individual values of day of reentrainment to the 8-h advanced LD cycle after following the control (black), exposure to NCRW at ZT12 (orange) and ZT21 (blue) conditions. Asterisks indicate significant differences determined by Tukey’s post hoc test following one-way repeated-measures ANOVA. * *P* < 0.05, *** *P* < 0.001.

Figure 2 illustrates representative double-plotted actograms of locomotor activity from mice subjected to an 8-h advance of the LD cycle following the control conditions and exposure to the NCRW at ZT12 and ZT21 conditions. Repeated measures ANOVA revealed a significant difference among the three conditions (*F* = 17.97, *P* < 0.0001). The number of days required for complete reentrainment of activity onset was significantly faster in mice exposed to NCRW at ZT12 (9.3 ± 1.5 days) compared with those in the control (10.9 ± 1.0 days, *P* = 0.011, Fig. 2G). In contrast, reentrainment in mice exposed to the NCRW at ZT21 (12.2 ± 1.6 days) was significantly slower than in the control (*P* < 0.0001, Fig. 2G).

Interestingly, closer inspection of individual mice exposed to the NCRW at ZT21 revealed notable variability. Four out of ten mice showed a much slower phase advance shift of activity onset for several days after the advance shift of the LD cycle (Fig. 2D), or some activity bouts appeared to show phase delays (Fig. 2E, F) despite the LD cycle being phase advanced, a phenomenon resembling antidromic reentrainment. To investigate this behavior, mice exhibiting antidromic reentrainment (*n* = 4) were compared with those showing orthodromic reentrainment (*n* = 6) following the exposure to NCRW at ZT21. Circadian profile of locomotor activity (3‑h bins) was examined for both the baseline period (10 days prior to the exposure to NCRW session) and the exposure to NCRW session (19 days). During the baseline period, mice that later showed antidromic reentrainment displayed significantly lower activity during the early to middle portion of the dark (active) phases (ZT12, ZT15, and ZT18) compared with mice showing orthodromic reentrainment (Fig. S4A). During the exposure to the NCRW session, similar trends were observed, but differences did not reach statistical significance (Fig. S4B). The total number of wheel revolutions during the NCRW session did not differ between the two groups (Fig. S4C).,,

### Experiment 3. Light-induced phase shift of activity onset and offset

**Fig. 3:**
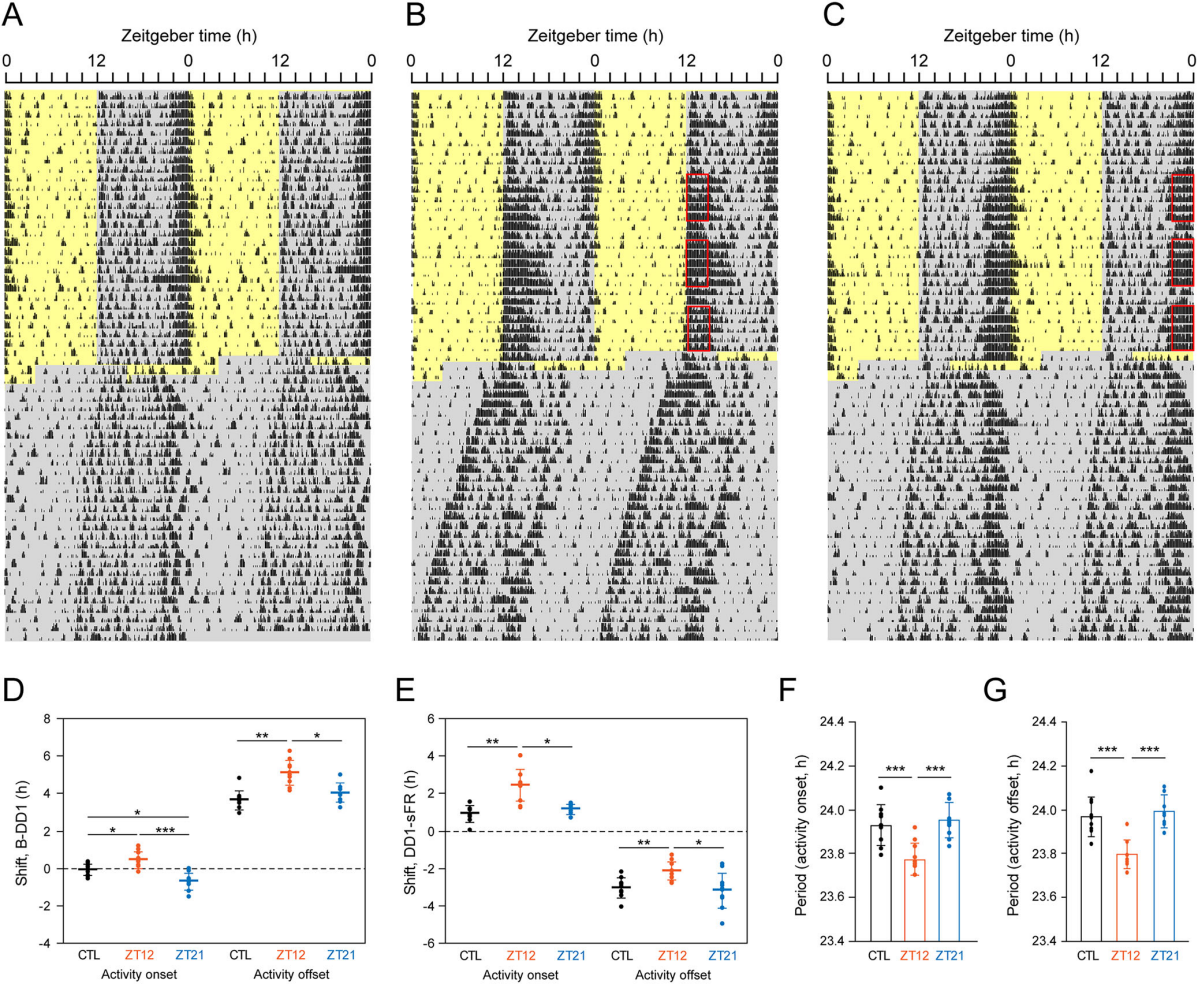
Representative actograms of locomotor activity from mice exposed to a new cage with a running wheel (NCRW) under a light-dark (LD) cycle, followed by a single 8-h advanced LD cycle, and released into constant darkness (DD). Representative double-plotted actograms of locomotor activity from the same mouse under three different conditions during an LD cycle: control (**A**), exposure to NCRW at ZT12 (**B**) and exposure to NCRW at ZT21 (**C**). Red squares indicate the 3-h NCRW exposure periods at ZT12-ZT15 (**B**) or ZT21-ZT0 (**C**). Yellow and gray areas indicate light and dark periods, respectively. Means phase shifts of activity onset and offset from the baseline day to the first day in DD (B-DD1, **D**) and from the DD1 to the transition day of steady-state free-run (DD1-sFR, **E**). Mean free-running period of activity onset (**F**) and offset (**G**) in DD. Small circles represent individual mice, and horizontal bars indicate the mean ± SD. Asterisks indicate significant differences determined by post hoc Tukey’s post hoc test following one-way repeated-measures ANOVA. *P < 0.05, **P < 0.01, ***P < 0.001.

Figure 3 illustrates representative double-plotted actograms (Fig. 3A–C) in mice subjected to a single 8-h advance of the LD cycle and then released into DD, following control conditions and exposure to the NCRW at ZT12 and ZT21.

Upon release into DD after single 8-h advance of LD cycle, the activity onset and offset on the first day of DD exhibited differential phase shifts (Fig. 3D, E). Phase shifts were compared with the differences in the activity phases (activity onset and offset) between the baseline (B) and the first day of DD (B-DD1; Fig. 3D), and between DD1 and the day on which the steady-state free-running period began (DD1-sFR; Fig. 3E). Activity onset showed a modest shift from baseline (B), whereas activity offset was markedly advanced, indicating alpha compression. Repeated measures ANOVA revealed a significant difference in the phase shift of B-DD1 among the three conditions (activity onset: *F* = 20.03, *P* = 0.0008; activity offset: *F* = 14.13, *P* = 0.0005). From the baseline to first day of DD (B-DD1), the activity onset was slightly but significantly advanced in mice exposed to NCRW at ZT12 (0.44 ± 0.44 h, *P* = 0.026) and significantly delayed in those exposed at ZT21 (−0.71 ± 0.45 h, *P* < 0.0001) compared with control condition (−0.07 ± 0.30 h) (Fig. 3D). The phase shift of activity offset (B-DD1) showed a significantly large advance in mice exposed to NCRW at ZT12 (5.09 ± 0.66 h) compared with control (3.64 ± 0.50 h, *P* = 0.0043) and NCRW at ZT21 (4.04 ± 0.49 h, *P* = 0.012) (Fig. 3D).

Following release into DD, phase shifts in activity onset and offset progressed through two distinct stages: an initial rapid shift, followed by a slower secondary shift and steady-state free-running. During the rapid phase shift, activity onset advanced, whereas activity offset was delayed, resulting in alpha decompression. The magnitude of phase advance in activity onset from DD1 to the day of the secondary shift (DD1–sFR) was significantly greater in mice exposed to the NCRW at ZT12 (2.45 ± 0.87 h) compared with control (0.91 ± 0.46 h, *P* = 0.0065) and exposure at ZT21 (1.15 ± 0.29 h, *P* = 0.0039) (Fig. 3E). In contrast, the phase delay in activity offset (DD1–sFR) was significantly smaller in mice exposed to NCRW at ZT12 (−2.14 ± 0.49 h) than in the other two conditions (control: −3.04 ± 0.52 h, *P* = 0.0293; exposure to NCRW at 21: −3.19 ± 0.95 h, *P* = 0.0227; Fig. 3E).

The free-running period of activity onset and offset, measured after the establishment of a steady-state phase relationship was significantly shortened in mice exposed to the NCRW at ZT12 (activity onset: 23.77 ± 0.09 h, Fig. 3F; activity offset: 23.80 ± 0.06 h, Fig. 3G) compared with control (activity onset: 23.93 ± 0.09 h, *P* < 0.0001, Fig. 3F; activity offset: 23.97 ± 0.09 h, *P* < 0.0001, Fig. 3G) and the exposure to NCRW at ZT21 (activity onset: 23.95 ± 0.08 h, *P* < 0.0001, Fig. 3F; activity offset: 23.99 ± 0.08 h, *P* < 0.0001, Fig. 3G). The free-running periods of activity onset and offset in the control condition were not significantly different from those in mice exposed to the NCRW at ZT21.

To clarify the timing and implementation of the crossover design, the total duration of the experimental period for each mouse was 195–200 days (approximately 6.5 months) in Experiment 1, 205 days (approximately 7 months) in Experiment 2, and 210-225 days (approximately 7 months) in Experiment 3. All three conditions (ZT12, ZT21, and Control) were completed within this timeframe (Fig. S1). The two experimental sequences (ZT12 → ZT21→Control and ZT21 → ZT12→Control) are illustrated in Fig. S1, and complete actograms covering the entire experimental period for each mouse are presented in Figs. S2, S3, and S5. Mice were 2–4 months old at the start of the experiments, and completion of all three conditions within 7 months resulted in an estimated age of approximately 9–11 months at the end of the study.

Post hoc power analyses were conducted for all major comparisons. Across experiments, the time‑dependent effects of NCRW on phase shifts, free‑running periods, and activity onset/offset dynamics were associated with large effect sizes (Cohen’s *d*), providing adequate statistical power with the sample sizes used (Table S1).

### Number of wheel revolutions during exposure to NCRW at ZT12 and ZT21

**Fig. 4:**
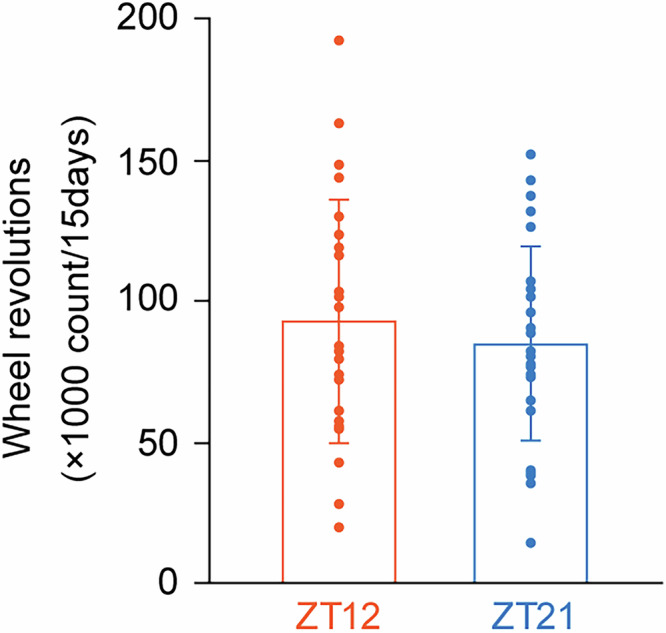
Wheel revolutions during exposure to new cage with a running wheel (NCRW) session at ZT12 and ZT21. Mean and individual values for the total wheel revolutions during the NCRW session at ZT12-15 (orange) and ZT21-ZT0 (blue). Small circles represent individual mice, and bars indicate the mean ± SD in mice exposed to NCRW at ZT12-ZT15 (orange) and ZT21-ZT0 (blue), respectively.

In all three experiments, mice (*n* = 28) were exposed to NCRW at ZT12 and ZT21 for a total of 15 days. Data from one mouse were excluded due to a mechanical issue, leaving 27 mice for analysis. A two-way repeated-measures ANOVA revealed no significant interaction between days of exposure (first, second, and third 5-day sessions) and time of exposure to the NCRW (ZT12 vs. ZT21) (*F*(2, 52) = 2.378, *P* = 0.1028). The total number of wheel revolutions over the 15-day exposure period did not differ significantly between ZT12 (92,730 ± 42,785 counts/3 h) and ZT21 (84,806 ± 34,693 counts/3 h) (*t* = 1.071, *P* = 0.294) (Fig. 4). Thus, the timing of NCRW exposure did not affect the overall amount of wheel-running activity.

## Discussion

This study aimed to examine whether the timing of exercise induced by exposure to an NCRW under a LD cycle alters the coupling strengths between E and M oscillators, which separately regulate activity onset and offset of circadian behavioral rhythms in nocturnal rodents^13–15^.

In Experiment 1, the free-running period of activity onset was significantly shortened when the exposure to NCRW occurred at ZT12 compared with control and exposure at ZT21. Mistlberger and Holmes (2000) reported that mice allowed to run on an RW in the home cage from later light period (ZT11) to mid-dark period (ZT18) under an LD cycle exhibited a shorter free-running period under DD than those allowed to run from the mid-dark period (ZT18) to late light period (ZT11)^12^. Although that study assessed exercise by locking or releasing the running wheel within the home cage, our results on the free-running period are consistent with these findings. Additional evidence indicates that the NCRW paradigm exerts effects on the circadian system that differ qualitatively from wheel locking or wheel unlocking procedures. Regarding nonphotic entrainment of circadian behavioral rhythms by timed exercise under DD^4,6,23^, daily exposure to the NCRW presented with a 24‑h period could entrain circadian locomotor activity rhythm and induced clear anticipatory activity prior to the NCRW exposure, and appeared the so-called after-effect of entrainment^6^. In contrast, timed exercise using wheel‑locking/unlocking protocols could also entrain circadian behavioral rhythms but failed to induce anticipatory activity or after-effect^4,23^. Moreover, NCRW exposure produced a pronounced after‑effect following termination of the exposure schedule, with free‑running periods shifting toward 24 h after entrainment—an outcome indicative of altered coupling force between E and M, which was not observed in wheel‑locking/unlocking protocols.

Several lines of evidence indicate that the NCRW exposure provides a uniquely potent nonphotic cue. The NCRW paradigm combines two interventions; voluntary wheel-running activity (exercise) and exposure to a novel environment (stress). We systemically examined phase shifts of mouse behavioral rhythms in response to an 8-h phase-advanced LD cycle and the effects of behavioral interventions applied from advanced dark onset (mid-subjective day, ZT4): maintained in a home cage (HC), exposed to an RW in the home cage (HCRW), transferred to a new cage (NC), and exposed to a new cage with an RW (NCRW)^19^. Phase shifts were significantly larger in the NCRW group than in the other three groups. Furthermore, the number of wheel revolutions did not differ significantly between the HCRW and NCRW groups. These findings demonstrated that neither novelty‑induced arousal in a new cage nor voluntary wheel running alone is sufficient; rather, the combination of voluntary exercise and exposure to a novel environment is essential for generating strong nonphotic phase-shifting signals. In addition, in hamsters, exposure to the NCRW during late subjective night (ZT20-ZT23, ZT21-ZT0) under a skeleton photoperiod (LDLD 0.5:10:0.5:13) significantly lengthened the free-running period of wheel-running activity under DD^24^.

Building on these established nonphotic effects, the present study introduces a novel combination of exercise and light manipulation by examining the phase-shifting influence of timed NCRW exposure under a stable LD 12:12 cycle. Because the SCN is entrained by light under LD conditions, the E and M oscillators regulate the phases of activity onset and offset, and stable circadian behavioral rhythms emerge through coordinated interactions between these two oscillators.

By applying NCRW at two distinct circadian times, we found that exposure at ZT12 preferentially strengthened the inter-coupling strength of the E oscillator to the M oscillator, consistent with a shift in inter-coupling pulling the M oscillator toward the phase of the E oscillator. In contrast, exposure at ZT21 produced a weaker but detectable shift in the opposite direction, suggesting a modest enhancement of the inter-coupling strength of the M oscillator to the E oscillator. These asymmetric effects were also supported by comparisons with control conditions, indicating that the impact of NCRW at ZT21 was smaller than that observed at ZT12. This approach differed from most previous studies conducted under DD^6,11^ and provides new insights into how exercise timing modulates the E and the M oscillators' dynamics.

Regarding the functional properties of the E and M oscillators, some previous studies demonstrated the amount of phase shift in activity onset and offset, which is evaluated on the first cycle after the light pulse in terms of the immediate PRC^16–18^. In contrast, the steady‑state PRC is derived from the stable phase shift observed 3–5 days transient period after the light pulse, which shows a shape similar to that of the PRC for activity onset and offset. The immediate PRC for activity onset has only delay shifts, whereas the immediate PRC for activity offset has both delay and advance portions^16–18^. This difference indicates that the acute response to a light pulse differs between the E and M oscillator outputs, even though their steady‑state PRCs converge to a similar form^17^.

Based on the shape of immediate PRC of activity onset and offset, free-running periods of E and M oscillators are suggested to be *τ* < 24 h and *τ* > 24 h, respectively. Furthermore, the steady-state free-running period under DD is thought to be determined by the balance of the inter-coupling strengths between E and M oscillators, which was successfully predicted by computer simulations^13^.

In the present study, a shortened free-running period of activity onset was observed in mice exposed to NCRW at ZT12. The PRC to a single NCRW exposure has a large phase-advance portion occurs in the middle of the subjective day and phase-delay portion that occurs in the late subjective night^5,25^. Exposure to NCRW at ZT12 induced a small phase advance shift, whereas the exposure at ZT21 induced a large phase delay shift. Therefore, the shortened free‑running period observed after NCRW at ZT12 may reflect the cumulative after-effect of daily small phase advance shifts^25^, rather than the consequence of a single day of exposure at ZT12. Another hypothesis is that NCRW exposure at ZT12 might have strengthened the oscillation of the E oscillator, which has a shorter intrinsic period, thereby altering the inter-coupling strength of the E oscillator to the M oscillator and shortening the overall rhythm^26^. In contrast, the free-running period after the NCRW exposure at ZT21 did not differ from that of the control condition, suggesting weaker effects on the M oscillator.

In Experiment 2, the reentrainment to an 8-h advanced LD cycle was accelerated by NCRW exposure at ZT12 but decelerated by NCRW exposure at ZT21. Based on immediate PRCs to light^16–18^, an 8-h advanced LD cycle does not provide photic input that induces phase advances in the immediate PRC of activity onset (E oscillator), whereas the immediate PRC of activity offset (M oscillator) is expected to elicit a large phase advance shift of activity offset. Therefore, the facilitation of reentrainment of activity onset by NCRW exposure at ZT12 is unlikely to be explained solely by light-induced phase advance shift, but rather by shortening of the E oscillator period and/or increase of the inter-coupling strength of the E oscillator to the M oscillator. Conversely, the decelerated reentrainment following the NCRW exposure at ZT21 may be due to the increase of inter-coupling strength of the M oscillator to the E oscillator, thereby interfering with phase advance shift of the activity onset.

In addition to the overall delay in reentrainment at ZT21, a subset of mice exhibited antidromic reentrainment, suggesting that individual differences in oscillator dynamics may modulate the direction of phase adjustment under these conditions.

Exploratory analyses revealed that mice showing antidromic reentrainment exhibited reduced activity during the early portion of the active phase under baseline conditions (Fig. S4A), despite comparable locomotor activity (Fig. S4B) and wheel revolutions (Fig. S4C) during the NCRW exposure sessions. Rather than reflecting differences in exercise engagement, this pattern may indicate weaker intrinsic output of the E oscillator to the M oscillator. Under this interpretation, NCRW exposure at ZT21—a time when nonphotic stimuli are thought to preferentially modulate inter-coupling strength of the M oscillator to the E oscillator—may further amplify the M oscillator influences in these individuals, increasing susceptibility to antidromic reentrainment. Although preliminary and based on a small number of mice (*n* = 4), these observations raise the possibility that individual differences in baseline activity organization, rather than exercise intensity (wheel revolutions), contribute to variability in the type of reentrainment.

In Experiment 3, the immediate phase shifts (B–DD1) following a single 8-h advanced LD cycle were larger in mice exposed to NCRW at ZT12 than in either the control or the NCRW exposure at ZT21. The large phase advance of activity onset was consistent with the results of Experiment 1, which demonstrated a shortened free-running period following exposure to NCRW at ZT12. Notably, activity offset also showed a large phase advance in the NCRW exposure at ZT12 condition (Fig. 3D). The advanced dark period overlaps with a delay portion in the immediate PRC both activity onset and activity offset^16–18^, suggesting that the large advance shift of activity offsets may be due to enhanced inter-coupling strength of the E oscillator to the M oscillator. As a result of this increased inter-coupling strength, the phase of the M oscillator was produced by NCRW exposure at ZT12. During the subsequent transient phase (DD1–sFR), the activity onset advanced further, whereas delays in activity offset were attenuated under the NCRW exposure at ZT12. Taken together, the results of Experiments 2 and 3 suggest that the exposure to NCRW at ZT12 enhances the inter-coupling strengths of the E oscillator to the M oscillator, thereby facilitating phase advance in activity onset while stabilizing activity offset.

Regarding the neural pathway involved in the effects of NCRW exposure on the E and M oscillators, voluntary wheel running induced by NCRW exposure likely elevated arousal levels in the brain, which might feed back to the SCN through multiple afferent pathways. Previous studies have implicated serotonergic inputs from the raphe nuclei^8^, NPY-containing neurons from the intergeniculate leaflet^8,27^, and orexinergic inputs from the lateral hypothalamus^28^, to the SCN are involved in the nonphotic phase shifting of circadian behavioral rhythms. For example, lesions of serotonergic and NPY-containing neurons abolish exercise-induced changes in the free-running period^8^, and SCN slices respond to serotonin with phase advances resembling nonphotic PRCs^28^. Similarly, NPY injections into the SCN induce phase shifts in the circadian rhythm of electrical activity that parallel nonphotic behavioral PRCs^29^. In addition, orexin signaling enhances the SCN clock-resetting effects of NPY, a neurochemical correlate of arousal^30^. Together, these findings suggest that exposure to NCRW enhances afferent inputs to the SCN via these systems, thereby altering the coupling strengths between the E and the M oscillators and modulating their responsiveness to light.

The present study has several limitations. First, only male mice were used. Recent study has reported marked sex differences in nonphotic entrainment and phase shifting induced by timed exposure to NCRW under DD^25^, suggesting that biological sex is an important variable in nonphotic phase resetting. Accordingly, the time-dependent effects observed here may differ in female mice. Future studies should therefore directly examine whether modulation of coupling strengths between E and M oscillators by timed exercise differs between sexes. Second, we did not assess feeding behavior, metabolic state, or hormone secretion (e.g., corticosterone, testosterone) during daily exposure to NCRW. These factors are known to influence the phase and period of circadian behavioral rhythm^31–34^. Future studies should test whether these factors influence the E and M oscillator coupling and circadian rhythms. In addition, the mice used in this study were 2–4 months old at the start of the experiments, and all three conditions were completed within 7 months, resulting in an estimated age of 9–11 months at the end of the study. This age range is well below the threshold at which age‑related deterioration of circadian rhythms becomes prominent. Previous studies have shown that clear aging effects such as lengthening of the free‑running period, reduced activity levels, and delayed reentrainment to advanced LD cycles typically occur in mice older than 18 months^35–37^. Future studies using aged mice will be important to determine whether the effects of timed exercise on circadian behavioral rhythms observed here are preserved or altered in older animals. Although this study suggests that exposure to NCRW modifies the coupling strengths between the E-M oscillators based on the phase shifts in behavioral activity onset and offset, further studies are required to directly evaluate how exposure to NCRW at different times of day affects the E-M oscillator coupling at the molecular level. Such analyses should include measurements of molecular clock markers (e.g., *mPer1* and *mPer2* gene expression, *Per1-luc*, PER2::LUC, and cFos expression) in the anterior and posterior regions of the SCN at the single-cell resolution. In addition, the neuropeptides, arginine vasopressin (AVP) and vasoactive intestinal polypeptide (VIP), are well established as key regulators of circadian rhythms stability within the SCN and in overt behavioral rhythms^23,38–40^. Assessing how timed NCRW exposure influences the neural signaling within the SCN will be an important focus of future studies. Additionally, the minimum duration of NCRW exposure required to alter circadian behavioral rhythms and the coupling strength between the E and M oscillators remains unclear, and systematic variation of exposure duration should be tested. Although the sample sizes used in this study were modest, post hoc power analyses indicated that the primary effects of NCRW exposure at two different times of day (ZT12 and ZT21) were associated with large effect sizes and were detected with sufficient statistical power (Table S1). Nevertheless, increasing the number of animals in future studies would further strengthen the generalizability of the present findings.

In conclusion, this study demonstrated that exposure to NCRW under an LD cycle alters the free-running period and light responsiveness of circadian rhythms in a time-dependent manner, likely through modulation of E-M oscillator coupling. These results suggest that combined exercise and light-based interventions may provide novel strategies for managing shift work, jet-lag, and circadian rhythm-related sleep disorders.

## Methods

### Animals

A total of 28 adult male C57BL/6J mice (CLEA Japan Inc., Tokyo, Japan) were used in this study. At the beginning of the experiments, the mice were 2–4 months old and weighed 25–28 g. Before experimentation, mice were bred and reared with three to four littermates in polycarbonate cages (17 × 29 × 13 cm^3^) in our institutional animal facility under a 12:12 h light–dark (LD) cycle (light intensity: ~300 lux at cage level), with a constant ambient temperature of 22–24 °C and relative humidity of 50–60%. Food and water were available ad libitum throughout the study.

At the start of the experiments, the mice were transferred to individual cages (15 × 25 × 15 cm^3^) equipped with a running wheel (10‑cm diameter) and allowed to acclimate to the wheel for two weeks. After the habituation period, animals were moved to a light‑tight chamber (40 × 50 × 30 cm^3^) and individually housed in cages without a running wheel. Light intensity, temperature, and humidity inside the chamber were maintained at levels equivalent to those in the animal facility.

### Experimental protocol

#### Experiment 1: phase angle of entrainment to the LD cycle and free-running period under DD

Eight mice were initially maintained under a 12:12 h LD cycle for at least 10 days. Subsequently, they were exposed to the NCRW for 3 h (11.0-14.0 h local time) daily, starting at ZT12 (dark onset; *n* = 4), or ZT21 (3 h before light onset; *n* = 4). To minimize potential masking effects of noise associated with wheel‑running activity and cage exchange (e.g., opening and closing the chamber door) on the circadian behavioral rhythms, the LD cycle was adjusted so that all NCRW sessions occurred at the same clock time (11.0–14.0 h). Figure S1 illustrates the schematic experimental protocols, in which the two NCRW conditions were administered in a crossover design (Figs. S1A and S1B). The NCRW exposure protocol followed our previous studies^6,11,22^. Briefly, at the beginning of each exposure session, mice were transferred from their home cage (without a running wheel) to a novel cage equipped with a running wheel. Cage exchange was performed outside the recording chamber under dim red light (<0.1 lx), and mice were returned to the chamber within 30 s. During the 3-h exposure period, mice were allowed to run voluntarily on the wheel. After each session, mice were returned to their home cage. This schedule was conducted for five consecutive days, followed by a 2-day rest period, and repeated weekly for three weeks (15 days total). Freshly cleaned cages and running wheels were used for each daily session. After the final exposure, mice were released into constant darkness (DD) for three weeks to assess free-running rhythms. Subsequently, all mice were reentrained to the 12:12 h LD cycle and subjected to the same NCRW exposure protocol at the opposite time of day (ZT12: *n* = 4; ZT21: *n* = 4) following a crossover design, so that each mouse experienced both NCRW exposure at ZT12 and ZT21 conditions. In the control condition, mice remained in their home cages without access to a running wheel for at least 3 weeks, followed by an additional three weeks in DD.

#### Experiment 2: reentrainment of circadian rhythms to an 8-h advance of the LD cycle

Ten mice were maintained under a 12:12 h light–dark (LD) cycle for at least 10 days before the start of the experiment. As in Experiment 1, all mice first completed the two NCRW conditions (ZT12 and ZT21), followed by the control condition at the end. After each intervention (NCRW at ZT12, NCRW at ZT21, or control), the dark onset of the LD cycle was advanced by 8 h by shortening the 12 h light period to 4 h. The mice were maintained under the advanced LD cycle for 3 weeks to evaluate the reentrainment of circadian behavioral rhythms. The experimental protocol for Experiment 2 is illustrated in Figs. S1C and S1D.

#### Experiment 3: light-induced phase shift following a single cycle of an 8-h advanced LD schedule and released into DD

During reentrainment to an 8-h advanced LD cycle in Experiment 2, it was difficult to accurately assess phase shifts in activity offset due to masking effects from the advanced light phase. To determine whether timed exposure to NCRW under LD conditions alters light-induced phase shifts in both activity onset and offset, an experimental protocol was designed to minimize the masking effects of light.

Ten mice first completed the two NCRW conditions (ZT12 and ZT21) in a crossover design, followed by the control condition at the end, as in Experiments 1 and 2. Immediately after each intervention, mice were exposed to a single cycle of an 8‑h advanced LD schedule, achieved by advancing the onset of darkness. Unlike Experiment 2, in which mice remained under the advanced LD cycle, mice in Experiment 3 were released into DD after the single cycle of the 8-h advanced LD schedule. They remained in DD for 3–4 weeks to evaluate light-induced phase shifts and free-running periods in both activity onset and offset. Afterward, all mice were reentrained to the LD cycle for at least two weeks before proceeding to the next intervention. The experimental protocol for Experiment 3 is illustrated in Figs. S1E and S1F.

In each Experiment (Experiments 1–3), the control condition was conducted last, followed by two NCRW exposure sessions at ZT12 and ZT21. Completing all three experimental conditions required approximately 195-225 days (7 months; Figs. S2, S3, and S5). As noted, mice were 2–4 months old at the start of the experiments and 9–11 months old by the end. The control condition was placed at the end so that any subtle age‑related changes during the experimental period would not affect the comparability of the data with previous studies examining animals older than 18 months^35–37^.

The number of mice used in each experiment (Experiment 1, *n* = 8; Experiment 2, *n* = 10; Experiment 3, *n* = 10) was based on our previous studies using NCRW exposure^19,20,22^, which consistently produced large phase-shifting effects. These sample sizes were sufficient to detect time-of-day effects of NCRW exposure on circadian behavioral rhythms, as confirmed by post hoc power analyses.

### Measurement of spontaneous locomotor activity and wheel-running activity

Locomotor activity in the cage was continuously monitored using a passive infrared sensor attached to the ceiling of the chamber. The sensor detected changes in the intensity of thermal radiation emitted from a mouse in association with body movement^41^. The number of movements was counted every 1 min and fed into a computer system (The Chronobiology Kit; Stanford Software Systems, Stanford, CA, USA). During NCRW exposure, the number of wheel revolutions was simultaneously recorded. Wheel revolutions were counted every 1 min using a microswitch sensor mounted on the platform holding the cage inside the chamber. The running wheel was vertical with a solid surface perforated by circular and lateral holes spaced 1.5 cm apart and had a diameter of 10 cm.

### Data analysis

Behavioral circadian rhythms were visualized as standard double-plotted actograms with 5-min bins, and activity onset and offset were estimated using CLOCKLAB software (Actimetrics, Evanston, IL, USA).

In Experiment 1, the phase angle difference (psi: ψ) between circadian behavioral rhythms and the LD cycle was calculated as the time difference between activity onset (or offset) and dark onset (or light onset), respectively. Free-running periods of activity onset and offset were determined by eye-fitted regression lines drawn through the respective time points over the final 10 days in constant darkness (DD).

In Experiment 2, reentrainment to the 8-h advanced LD cycle was assessed. Reentrainment was defined as the reestablishment of a phase angle difference between activity onset (or offset) and the new dark onset (or light onset) within 30 min, maintained for at least three consecutive days. The number of days required for full reentrainment was determined as the day on which both activity onset and offset met this criterion.

In Experiment 3, following release into DD after a single cycle of the 8-h advanced LD schedule, phase shifts exhibited two distinct stages: an initial rapid shift that gradually slowed, followed by a slower but steady shift. This two-step phase-shift pattern was previously demonstrated using a similar protocol^19^. Following that study^19^, the transition point between the fast and slow stages was defined as the separate free-run (sFR), which could differ between activity onset and offset. The day when both activity onset and offset began to free-run in parallel was defined as the steady-state free-run (FR). Thus, FR corresponded to the sFR of either onset or offset, whichever occurred later. Baseline activity onset and offset (denoted as B) were calculated by averaging values from the 10 consecutive days of the LD cycle prior to NCRW exposure, as activity rhythms were masked during the exposure period. Phase shifts were then calculated separately for activity onset and offset across two intervals between baseline and the first day in DD following the advanced LD cycle (B–DD1), and between DD1 and sFR (DD1–sFR).

### Ethical approval

All animal procedures were approved by the Animal Research Committee of Hokkaido University (Permission No. 18-0174) and were conducted in accordance with institutional guidelines for the care and use of laboratory animals.

### Statistical analysis

All data are expressed as mean ± standard deviation (SD). Time-series data were analyzed using one-way repeated-measures ANOVA, followed by Tukey’s post hoc test. Student’s *t* test was used for pairwise comparisons, and unpaired *t*-tests were used for comparisons between two independent groups. A *P*-value less than 0.05 was considered statistically significant. All statistical analyses were performed using GraphPad Prism version 8 (GraphPad Software, San Diego, CA, USA).

## Supplementary information

Supplementary Figure S1-5+Table S1

Supplementary Data.

## Data Availability

The datasets used and/or analyzed in the present study are available from the corresponding author on reasonable request.
