# Supplementary material for: Timed exercise modulates inter-coupling strength between evening and morning oscillators in mice
Source: NPJ Biol Timing Sleep. 2026 Mar 27;3:12. doi: 10.1038/s44323-026-00075-3 (PMC13031686; doi:10.1038/s44323-026-00075-3)
Supplement: Supplementary file 1 — Supplementary Figure S1-5+Table S1 [file 44323_2026_75_MOESM1_ESM.pdf]

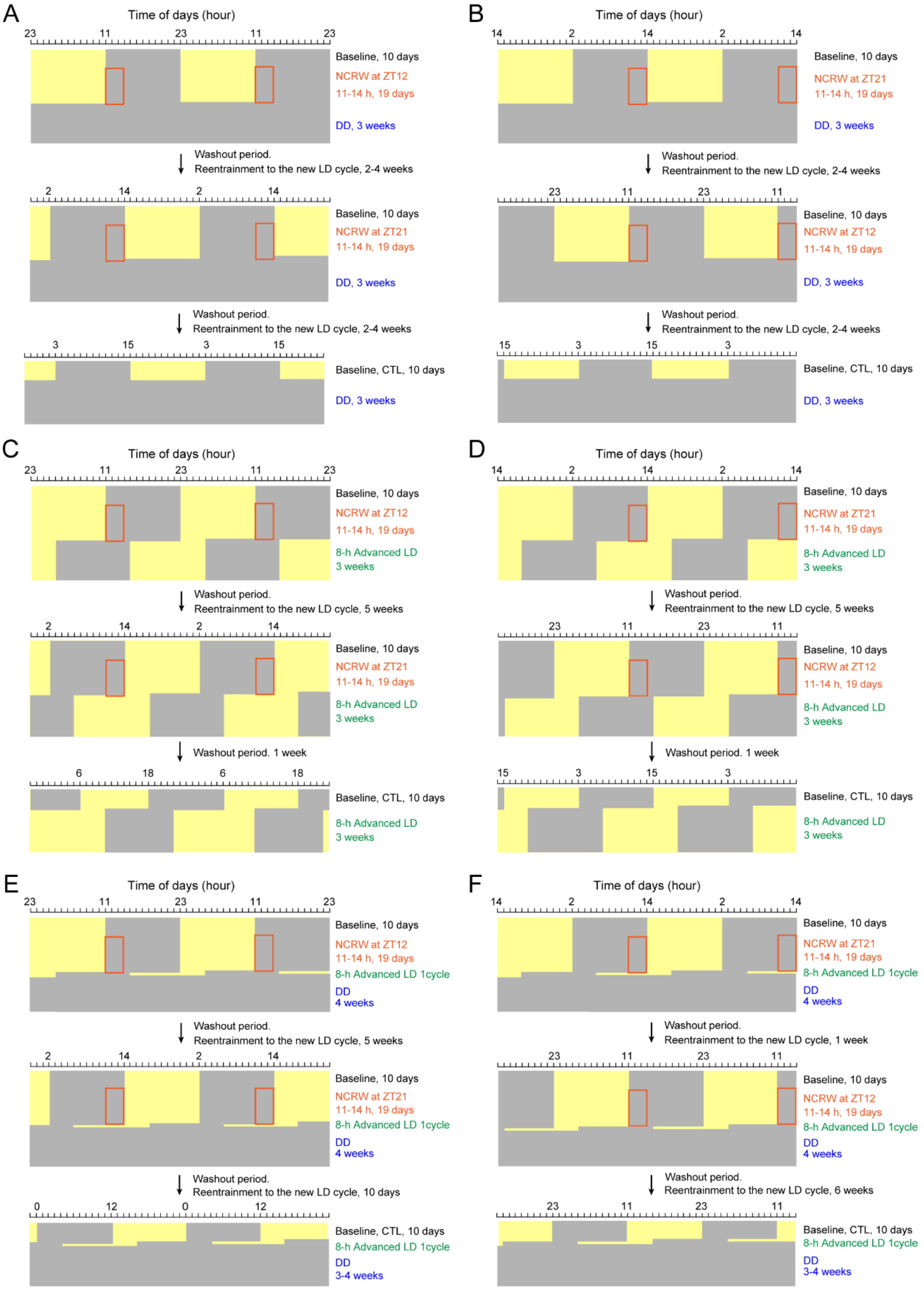
A
Time of days (hour)
23 11 23 11 23
Baseline, 10 days
NCRW at ZT12
11-14 h, 19 days
DD, 3 weeks
Washout period.
Reentrainment to the new LD cycle, 2-4 weeks
2 14 2 14
Baseline, 10 days
NCRW at ZT21
11-14 h, 19 days
DD, 3 weeks
Washout period.
Reentrainment to the new LD cycle, 2-4 weeks
3 15 3 15
Baseline, CTL, 10 days
DD, 3 weeks
B
Time of days (hour)
14 2 14 2 14
Baseline, 10 days
NCRW at ZT21
11-14 h, 19 days
DD, 3 weeks
Washout period.
Reentrainment to the new LD cycle, 2-4 weeks
23 11 23 11
Baseline, 10 days
NCRW at ZT12
11-14 h, 19 days
DD, 3 weeks
Washout period.
Reentrainment to the new LD cycle, 2-4 weeks
15 3 15 3
Baseline, CTL, 10 days
DD, 3 weeks
C
Time of days (hour)
23 11 23 11 23
Baseline, 10 days
NCRW at ZT12
11-14 h, 19 days
8-h Advanced LD
3 weeks
Washout period.
Reentrainment to the new LD cycle, 5 weeks
2 14 2 14
Baseline, 10 days
NCRW at ZT21
11-14 h, 19 days
8-h Advanced LD
3 weeks
Washout period. 1 week
6 18 6 18
Baseline, CTL, 10 days
8-h Advanced LD
3 weeks
D
Time of days (hour)
14 2 14 2 14
Baseline, 10 days
NCRW at ZT21
11-14 h, 19 days
8-h Advanced LD
3 weeks
Washout period.
Reentrainment to the new LD cycle, 5 weeks
23 11 23 11
Baseline, 10 days
NCRW at ZT12
11-14 h, 19 days
8-h Advanced LD
3 weeks
Washout period. 1 week
15 3 15 3
Baseline, CTL, 10 days
8-h Advanced LD
3 weeks
E
Time of days (hour)
23 11 23 11 23
Baseline, 10 days
NCRW at ZT12
11-14 h, 19 days
8-h Advanced LD 1cycle
DD
4 weeks
Washout period.
Reentrainment to the new LD cycle, 5 weeks
2 14 2 14
Baseline, 10 days
NCRW at ZT21
11-14 h, 19 days
8-h Advanced LD 1cycle
DD
4 weeks
Washout period.
Reentrainment to the new LD cycle, 10 days
0 12 0 12
Baseline, CTL, 10 days
8-h Advanced LD 1cycle
DD
3-4 weeks
F
Time of days (hour)
14 2 14 2 14
Baseline, 10 days
NCRW at ZT21
11-14 h, 19 days
8-h Advanced LD 1cycle
DD
4 weeks
Washout period.
Reentrainment to the new LD cycle, 1 week
23 11 23 11
Baseline, 10 days
NCRW at ZT12
11-14 h, 19 days
8-h Advanced LD 1cycle
DD
4 weeks
Washout period.
Reentrainment to the new LD cycle, 6 weeks
23 11 23 11
Baseline, CTL, 10 days
8-h Advanced LD 1cycle
DD
3-4 weeks

Figure S1. Experimental protocols.

Schematic representation of the experimental protocols for Experiment 1 (A, B), Experiment 2 (C, D), and Experiment 3 (E, F), which are plotted against clock time (hours). Panels on the left (A, C, E) and right (B, D, F) show the experimental protocols for mice first exposed to NCRW at ZT12 (left: A, C, E) or ZT 21 (right: B, D, F), respectively. Yellow and gray areas indicate the light and dark periods, respectively. Red squares indicate the 3-h NCRW exposure periods at ZT12 or ZT21.

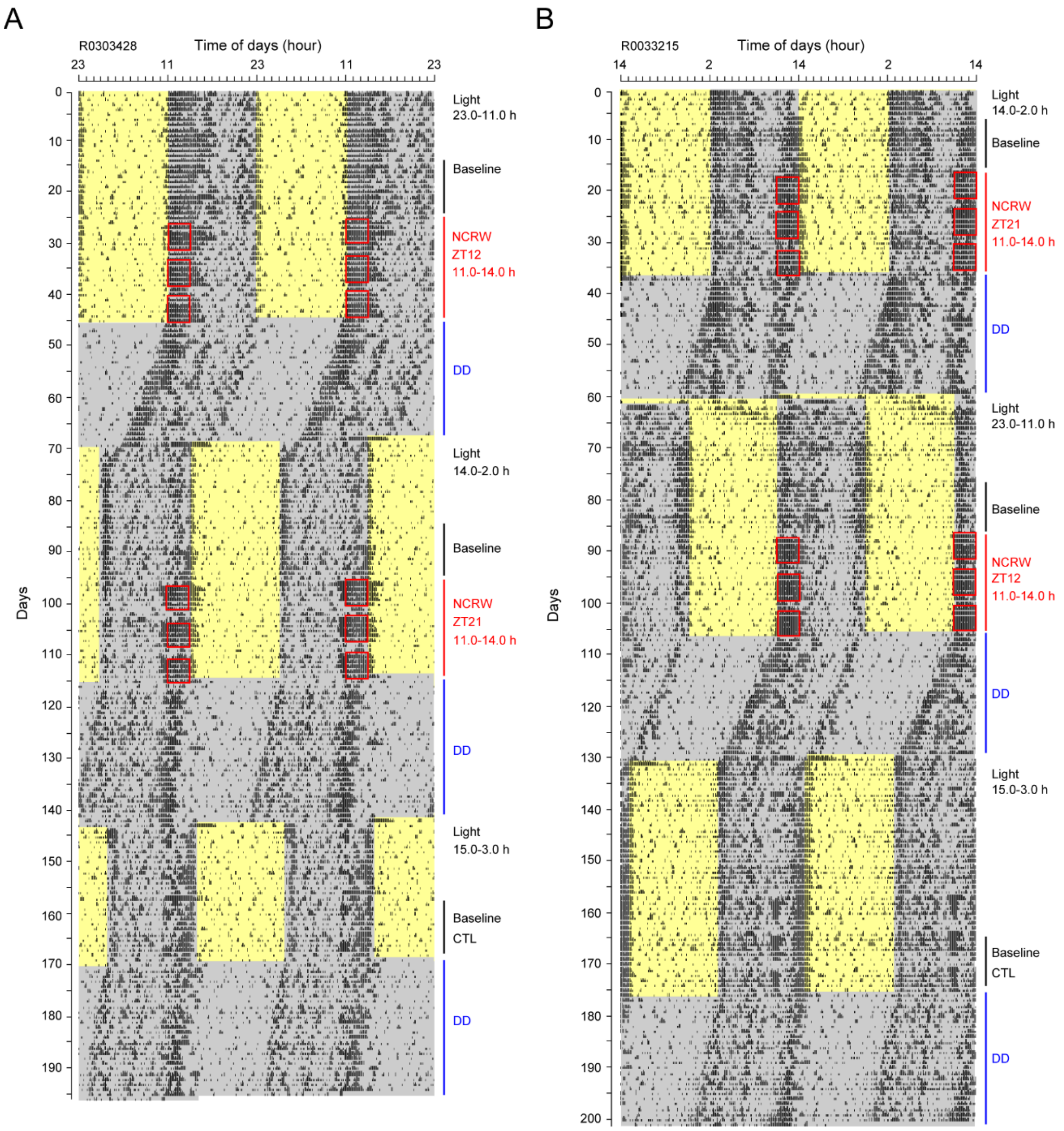

Figure S2. Representative double-plotted actograms of locomotor activity from Experiment 1 of individual mice first exposed to NCRW at ZT12 (A) or ZT21 (B).

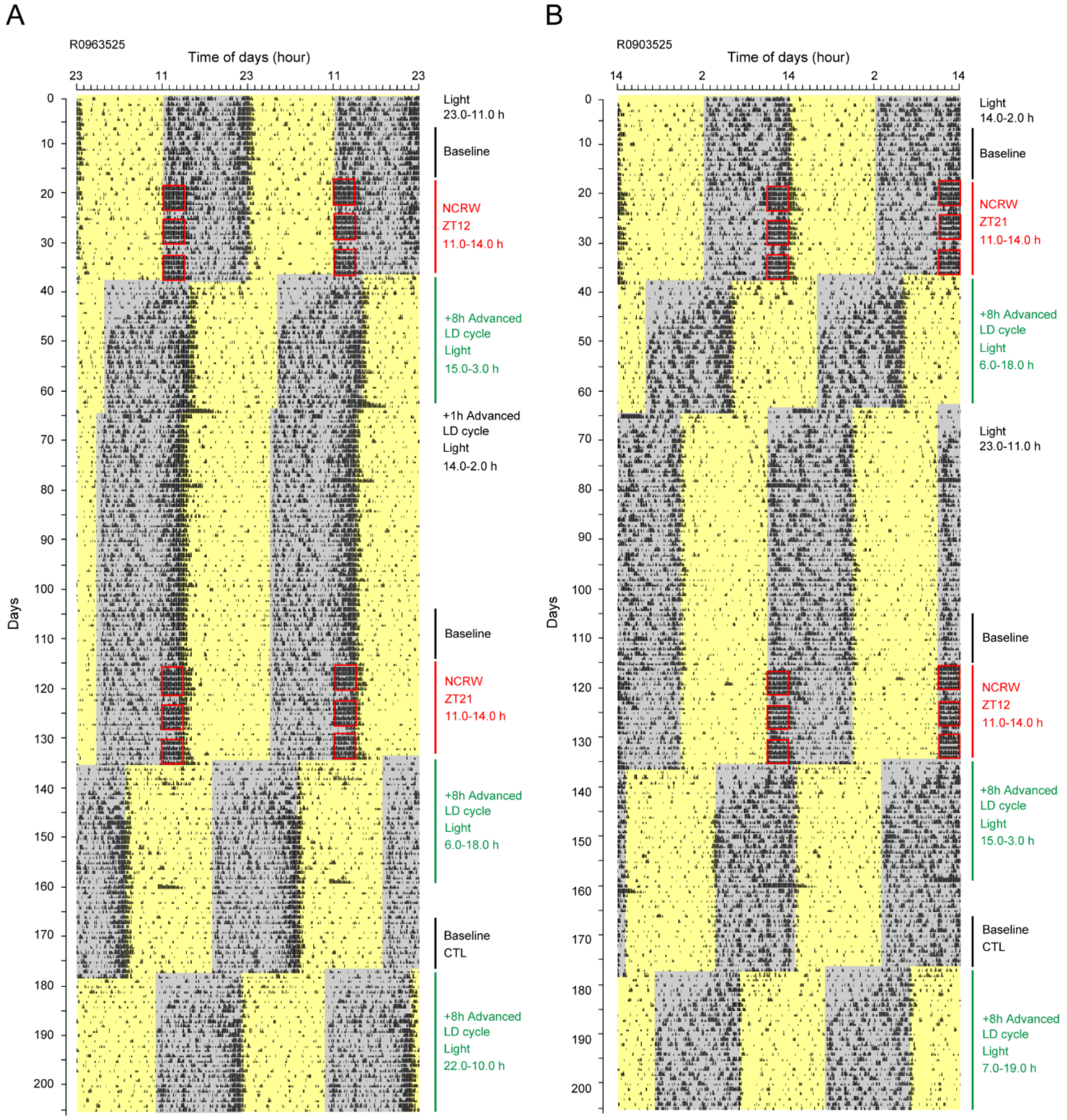

Figure S3. Representative double-plotted actograms of locomotor activity from Experiment 2 of individual mice first exposed to NCRW at ZT12 (A) or ZT21 (B).

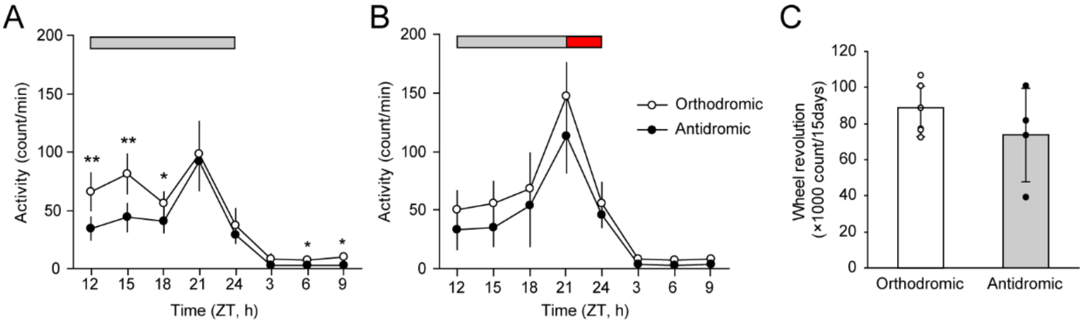

Figure S4. Circadian profiles of locomotor activity and wheel revolutions in mice from Experiment 2 showing orthodromic and antidromic reentrainment.

Panels A and B show the mean circadian profiles of locomotor activity in mice exhibiting orthodromic (open symbols) and antidromic (closed symbols) reentrainment. Data are plotted relative to the onset of the dark period of the LD cycle during the baseline period (A) and for 19 days during exposure to NCRW at ZT21 (B). Horizontal gray bars in A and B indicate the dark period of the LD cycle. The red area indicates the 3-h exposure to NCRW at ZT21. Asterisks indicate significant differences determined by two-way repeated-measures ANOVA followed by Tukey's post hoc test. * $P<0.05$, ** $P<0.01$. Panel C shows the mean total number of wheel revolutions (counts per 15 days) in mice exhibiting orthodromic (opened bars) and antidromic (gray bars) reentrainment. Small circles indicate values from individual mice.

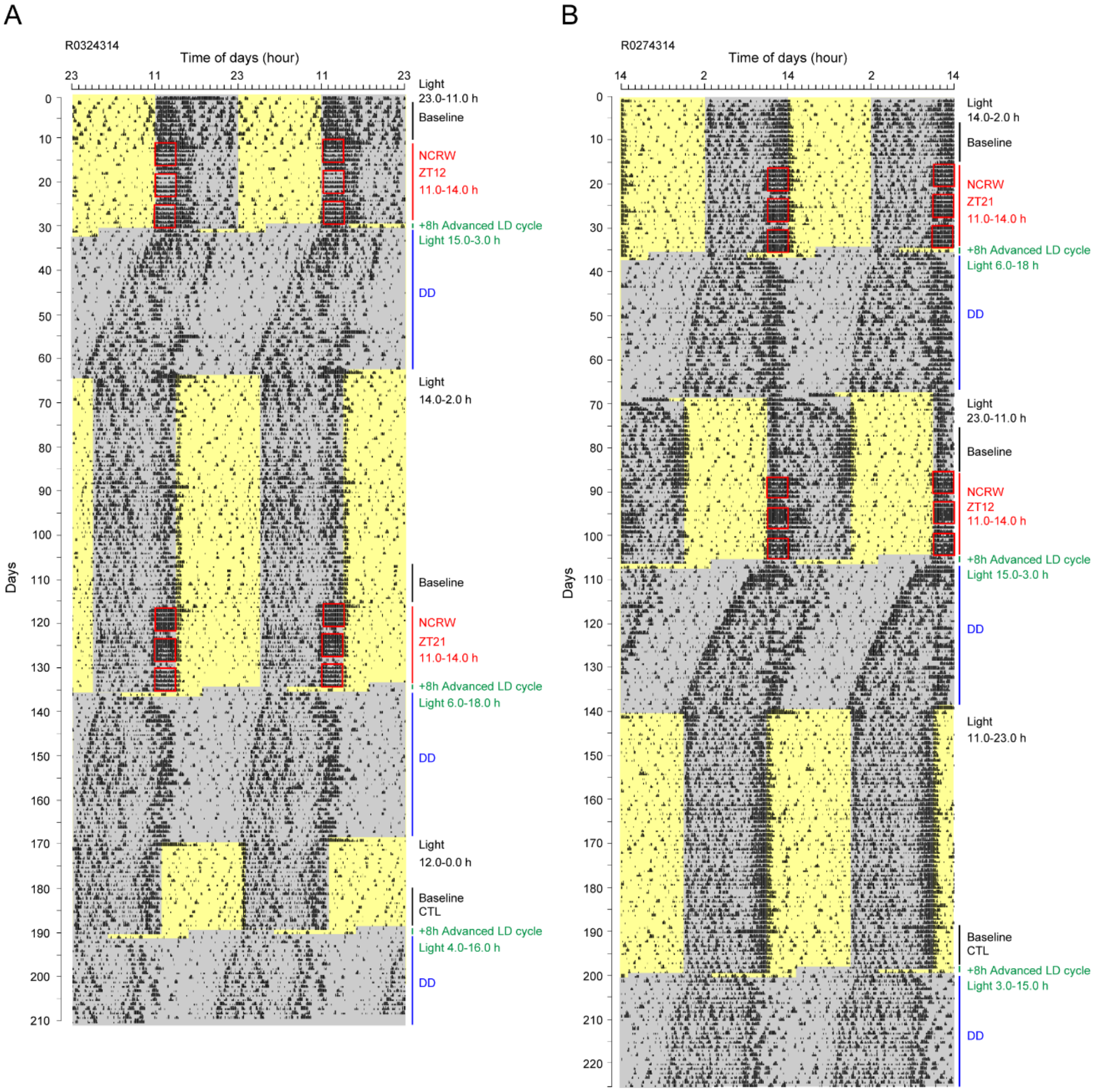

Figure S5. Representative double-plotted actograms of locomotor activity from Experiment 3 of individual mice first exposed to NCRW at ZT12 (A) or ZT21 (B).

Table S1. Summary of statistical significance, effect sizes, and power in the Experiments 1–3.

Experiment	Parameter	Comparison	p-value (Tukey)	Summary	Effect size (Cohen's *d*)	Power (approx.)	Interpretation
Exp 1 (N=8)	Period of activity onset	CTL vs ZT12	0.0093	**	>1.0	>0.80	Large effect
		CTL vs ZT21	0.7409	n.s	~0.2	~0.10	Very small effect
		ZT12 vs ZT21	0.0390	*	>1.0	>0.90	Large effect
	Period of activity offset	CTL vs ZT12	0.0443	*	>1.1	>0.87	Large effect
		CTL vs ZT21	0.9916	n.s	~0.22	~0.15	Small effect
		ZT12 vs ZT21	0.0351	*	>1.1	>0.90	Large effect
	Psi between light-offset and activity onset	CTL vs ZT12	0.0003	***	>2.5	>0.99	Very large effect
		CTL vs ZT21	0.4835	n.s	~0.77	~0.50	Moderate effect
		ZT12 vs ZT21	<0.0001	***	>2.5	>0.99	Very large effect
	Psi between light-onset and activity offset	CTL vs ZT12	0.5855	n.s	~0.9	~0.70	Large effect
		CTL vs ZT21	0.2276	n.s	~0.36	~0.20	Small–moderate effect
		ZT12 vs ZT21	0.7511	n.s	~0.9	~0.70	Large effect
Exp 2 (N=10)	Days of reentrainment	CTL vs ZT12	0.0105	*	1.22	~0.93	Large effect
		CTL vs ZT21	0.0385	*	0.88	~0.75	Large effect
		ZT12 vs ZT21	<0.0001	***	1.92	>0.99	Very large effect
Exp 3 (N=10)	Phase shift of activity onset (B-DD1)	CTL vs ZT12	0.0274	*	~1.0	~0.88	Large effect
		CTL vs ZT21	0.0073	**	~0.48	~0.30	Moderate effect
		ZT12 vs ZT21	<0.0001	****	~1.5	>0.99	Very large effect
	Phase shift of activity offset (B-DD1)	CTL vs ZT12	0.0002	***	~1.1	~0.90	Large effect
		CTL vs ZT21	0.3437	ns	~0.58	~0.40	Moderate effect
		ZT12 vs ZT21	0.0043	**	~1.47	>0.99	Very large effect
	Phase shift of activity onset (DD1-sFR)	CTL vs ZT12	<0.0001	****	>3.0	>0.99	Extremely large effect
		CTL vs ZT21	0.673	ns	~0.85	~0.73	Large effect
		ZT12 vs ZT21	0.0006	***	>2.0	>0.99	Very large effect
	Phase shift of activity offset (DD1-sFR)	CTL vs ZT12	0.0162	*	~1.7	>0.99	Very large effect
		CTL vs ZT21	0.8567	ns	~0.48	~0.30	Moderate effect
		ZT12 vs ZT21	0.0052	**	~1.56	>0.99	Very large effect
	Period of activity onset	CTL vs ZT12	<0.0001	****	>2.1	>0.99	Very large effect
		CTL vs ZT21	0.6899	ns	~0.67	~0.55	Moderate effect
		ZT12 vs ZT21	<0.0001	****	>2.1	>0.99	Very large effect
	Period of activity offset	CTL vs ZT12	<0.0001	****	>2.2	>0.99	Very large effect
		CTL vs ZT21	0.5361	ns	~0.65	~0.50	Moderate effect
		ZT12 vs ZT21	<0.0001	****	>2.5	>0.99	Very large effect
